# A Toxicogenomic Approach Reveals a Novel Gene Regulatory Network Active in In Vitro and In Vivo Models of Thyroid Carcinogenesis

**DOI:** 10.3390/ijerph16010122

**Published:** 2019-01-04

**Authors:** Carla Reale, Filomena Russo, Sara Carmela Credendino, Danila Cuomo, Gabriella De Vita, Massimo Mallardo, Francesca Pennino, Immacolata Porreca, Maria Triassi, Mario De Felice, Concetta Ambrosino

**Affiliations:** 1 IRGS, Biogem, Via Camporeale, 83031 Ariano Irpino, Avellino, Italy; carla.reale@biogem.it (C.R.); filomena.russo@biogem.it (F.R.); 2 Department of Molecular Medicine and Medical Biotechnologies, University of Naples “Federico II”, 80131 Naples, Italy; credendinosara@gmail.com (S.C.C.); gdevita@unina.it (G.D.V.); massimo.mallardo@unina.it (M.M.); 3 Department of Molecular and Cellular Medicine, College of Medicine, Texas A&M University, College Station, TX 77843, USA; cuomo@medicine.tamhsc.edu; 4 Department of Science and Technology, University of Sannio, via Port’Arsa 11, 82100 Benevento, Italy; 5 Department of Public Health, University of Naples “Federico II”, 80131 Naples, Italy; francesca.pennino@unina.it (F.P.); triassi@unina.it (M.T.); 6 Human Genetics, Wellcome Trust Sanger Institute, Hinxton CB10 1SA, UK; ip9@sanger.ac.uk; 7 IEOS-CNR, Via Pansini 6, 80131 Naples, Italy

**Keywords:** pesticides, TCDD, gene expression, thyroid cancer, cell survival

## Abstract

Epidemiological and experimental studies emphasize the link between environmental chemicals exposure and thyroid cancer. However, this association is strongly debated and the mechanisms of action of environmental thyroid carcinogens still need to be identified. The analysis of in vitro transcriptomic data developed to investigate the effects of chlorpyrifos on immortalized thyrocytes highlighted the impaired expression of genes involved in endodermal carcinogenesis. This endodermal carcinogenic gene-network (ECGN, including *Zfp36l2*, *Dmbt1*, *Ddit4*), was validated in cellular and mouse models of thyroid carcinogenesis, characterized by the constitutive activation of the mitogen-activated protein kinase (MAPK) pathway and in immortalized thyrocytes exposed to tetrachlorodibenzo-*p*-dioxin (TCDD) and chlorpyrifos (CPF). The mRNA levels of *Zfp36l2*, *Dmbt1* and *Ddit4* were increased in models characterized by MAPK activation or following TCDD exposure, whereas they were inhibited by CPF exposure. Overall, the ECGN transcripts identify a novel gene-regulatory network associated with thyroid carcinogenesis promoted by genetic mutation or by environmental carcinogens. The latter have opposite effects on the modulation of the ECGN transcripts according to their mechanisms of action in promoting carcinogenesis. Therefore, the analyses of ECGN might be helpful in discriminating compounds that promote cellular survival associated or not to proliferation of thyrocytes.

## 1. Introduction

Thyroid disrupting chemicals (THDCs) are endocrine disruptor compounds (EDCs) exerting their effects on the function and regulation of the thyroid tissue, especially during the early-life stages [1,2]. Among endocrine organs, the thyroid gland is highly susceptible to environmental pollutants that are considered the sole or contributory cause of thyroid cancer [3]. Rodent studies have shown that environmental chemicals, such as tetrachlorodibenzo-*p*-dioxin (TCDD), interfere with hypothalamus-pituitary thyroid (HPT) axis and thyroid hormone (TH) signalling and have been associated with thyroid cancer [4]. The mechanisms of such activity are strongly debated, although increased thyroid-stimulating hormone (TSH) levels and oxidative stress have been described as endogenous factors contributing to the rise in thyroid cancer incidence. Notably, both are increased after exposure to EDCs such as Bisphenol A (BPA) [5] or TCDD [6]. Furthermore, signalling pathways, deeply involved in thyroid carcinogenesis, are also deregulated by the same factors [7,8].

Several environmental EDCs exert THDC activity, mainly inducing hypothyroidism that is controversially suggested as a risk factor for thyroid cancer [9,10,11,12]. Epidemiological studies point to a positive association between pesticide use and hypothyroidism in both men and women [13,14], whereas only few papers reported an elevated risk of thyroid cancer associated with exposure to pesticides and related occupations among women only [15,16]. Although future prospective studies are warranted to investigate the association and identify potential causal agents, the link between thyroid dysfunction and pesticides exposure is suggested in different epidemiological [17,18] and experimental studies [19,20]. The identification of mechanisms of action of environmental pollutants on thyroid will help in solving the debate.

The traditional toxicology approach, based on long-lasting in vivo experiments, did not provide exhaustive information about the mechanisms of action of THDCs [21] in the impairment of the HPT axis and in thyroid carcinogenesis. Transcriptomics has been suggested as a powerful tool to identify the mechanisms underlying compound toxicity. This approach provides expression profiles of many hundreds of genes in a specific biological condition, which can help in understanding the related phenotype and molecular changes. In addition, pathway analysis allows for clustering of gene-expression data into relevant pathway maps based on their functional annotation and known molecular interactions.

Molecular mechanisms of thyroid carcinogenesis are well characterized [22] and the role of mitogen-activated protein kinase (MAPK) pathway has been established [23]. It has been shown that cAMP and Ras signalling simultaneous activation promotes apoptosis in thyrocytes and that a second event in surviving cells is able to induce loss of responsiveness to TSH and/or inactivation of apoptotic cascade, leading to clonal expansion [24].

We have used a toxicogenomic approach to reveal if environmental pollutants might promote thyroid carcinogenesis by already described or alternative pathways and by deregulating cellular processes such as proliferation and/or survival. Furthermore, we have identified mechanisms of such activity relying on the transcriptional or post-transcriptional regulation of gene expression, the latter being recognized as a major player in cancer development and progression.

Aiming to reveal the carcinogenic potential and mechanisms of action of pesticides exerting a THDC activity, such as chlorpyrifos (CPF), we analysed transcriptomic data previously obtained from immortalized thyrocytes (PCCL3) exposed to different doses of CPF [25]. In our previous analysis we observed the alteration of the expression of genes involved in hepatic carcinogenesis. Similar results came out from a study on FRTL-5, immortalized rat thyrocytes, exposed to BPA. Starting from these observations, we proposed that mechanisms of toxicity can be similar in organ shearing the developmental origin [26]. Since thyroid and liver are both derived from endoderm, we chose some of the genes potentially involved in liver carcinogenesis as components of a signature that might be applied to other endodermal organs, herein defined endodermal carcinogenic gene-network (ECGN). Up to published results, the selected genes were either playing a role in cancer development as *Dmbt1*, considered candidate tumor suppressor genes for several cancer types [27], or suppressors of mRNA translation and protecting from cancer development as *Cpeb2* [28].

The selected gene network is here investigated in different in vitro and in vivo models of thyroid cancer. The deregulation of ECGN was also evaluated in response to a well-established environmental carcinogen such as TCDD and the same CPF in different thyroid cell lines, by obtaining consistent results.

Overall, the reported finding shed light on a novel gene network involved in thyroid carcinogenesis, although with different mechanisms and roles in fully transformed conditions compared to the early step of cellular transformation.

## 2. Materials and Methods

Chlorpyrifos (Greyhound Chromatography F2057, Greyhound Chromatography, Birkenhead, UK) was dissolved in dimethyl sulfoxide at stock concentration of 500 mM. TCDD (Chemical Research 2000 s.r.l., Rome, Italy) dissolved in Dimethyl Sulfoxide (DMSO) at a concentration of 100 μM. Tg-rtTA-TetO-BrafV600E transgenic mice were fed with a 2500 mg/kg Doxycycline-supplemented food for one week in order to induce thyroid cancer.

### 2.1. Ethics Statement

Animal experiments were performed in accordance with the European Council Directive 2010/63/EU, following the rules of the D.Lgs 26/14, and procedures were approved by the Ethical committee of the Biogem Institute, Genetics Research “Gaetano Salvatore” (IRGS) and by the Italian Minister of Health. The ID numbers are: 1-2010 for CPF exposed animals and 13754-2014 for Tg-rtTA-TetO-BrafV600E mice.

### 2.2. Animals and Treatments

#### 2.2.1. Chlorpyrifos Exposure

Mice were kept under standard facility conditions and received water and standard diet (4RF21, Mucedola s.r.l., Milan, Italy) ad libitum. To allow the dosages of 1 mg/kg/day and 10 mg/kg/day CPF (Greyhound Chromatography F2057), the pesticides was added to the food at the concentrations of 44 and 4,4 respectively (prepared by Mucedola). We exposed pregnant CD1 dams to CPF (10 mg/kg/day, 1 mg/kg/day) until the weaning; after that the offspring (10 females for each condition) were directly exposed. After 6 months of treatment 4 mice/group were sacrificed and the blood and several organs were collected. Remaining animals were continuously treated till sacrifice (12 months) for blood and organ sampling. Mice were sacrificed by carbon dioxide inhalation.

#### 2.2.2. Doxycycline Treatment

Tg-rtTA-TetO-BrafV600E transgenic mice were kept under standard facility conditions at the animal facility of Department of Molecular Medicine and Medical Biotechnology in Naples. Animals received water and standard diet (4RF21 from Mucedola) ad libitum. In order to induce thyroid cancer, Tg-rtTA-TetO-BrafV600E transgenic mice were fed with a 2500 mg/kg Doxycycline-supplemented food for one week (custom formulated by Mucedola), then were sacrificed by carbon dioxide inhalation for organ sampling.

### 2.3. Cell Culture and Treatment

FRTL-5 cell line is an immortalized rat thyroid cell line mimicking normal differentiated thyrocytes; dependent from TSH for growth and differentiated functions, including iodide uptake and thyroglobulin and thyroperoxidase gene transcription. These properties of the cells were verified 1 week after thawing and cells were discarded every 12 passages (6 weeks). They were grown in Coon’s modified F12 medium (EuroClone S.p.A., Milan, Italy) supplemented with 5% newborn bovine serum (HyClone Laboratories Inc., Logan, Utah, USA) and six hormones mixture: insulin (Life Technologies), 10 μg/mL; hydrocortisone (Sigma Aldrich, Saint Louis, Missouri, USA), 10 nM; transferrin (Sigma Aldrich), 5 μg/mL; glycyl-L-histidyl-L-lysine acetate (Sigma Aldrich), 20 ng/mL; somatostatin (Sigma Aldrich), 10 ng/mL; and thyrotropin (Sigma Aldrich), 0.5 milliunits/mL.

Exponentially growing cells were exposed to TCDD diluted at 1 × 10^−9^ M or CPF 1 × 10^−9^ M in culture medium for 1 or 7 days. Controls were treated with vehicle alone.

FRTL-5-RasV12 cells are FRTL-5-derived cell line constitutively expressing an oncogenic form of H-Ras, bearing the V12 mutation [29]. These cells are de-differentiated and show several hallmarks of cancer cells, such as growth factor-independent proliferation, tumour development in nude mice and enhanced motility [30,31]. FRTL-5-RasV12 cells were cultured under the same culture conditions already described for parental FRTL-5 cells.

### 2.4. RNA Sequencing Data and Bioinformatics Analyses

RNAseq experiments were previously conducted on PCCL3 cell line and have been already described, also reporting details on cluster building approach [32].

Functional enrichment analysis was performed to understand the biological meaning of resulting deregulated genes for each cluster. Bioinformatics analyses was conducted by Ingenuity Pathway Analysis (IPA, QIAGEN Hilden, Germany). Over-represented biological and toxicological functions were found by Fisher exact test. Significant categories were selected by Benjamini and Hochberg method, for adjusting corrected *p*-values.

### 2.5. RNA Extraction and Quantitative Reverse Transcription PCR

Total RNA, from tissue and cells, was isolated with TRIzol reagent (Invitrogen, Carlsbad, CA, U.S.A.) according to the manufacturer’s instructions. For quantitative reverse transcription PCR, 1 µg total RNA was reverse-transcribed (QuantiTect Reverse Transcription Kit, QIAGEN or SensiFAST cDNA synthesis kit, Bioline, London, UK), according to the manufacturer’s instructions. Design of primers and qPCR were accomplished using NCBI Primer Blast and Fast SYBR Green Master Mix (Applied Biosystems with Applied Biosystem QuantStudio 7 Flex System) respectively. Primer sequences are listed in Supplementary Material (see Supplementary Table S2 online). Data were normalized by the level of internal control *Gapdh* expression in each sample. The 2^−ΔΔCt^ method was used to calculate relative expression changes.

### 2.6. Protein Extraction and Western Blotting

Whole extracts were prepared and quantified as already reported [33]. Rabbit antibodies against ZFP36L2 (PAB17366), pERK (CST, #9101), and ERK1/ERK2 (CST, #9102) and mouse antibodies against DMBT1 (sc-514566), DDIT4 (sc-376671), and β-actin (A5316-Sigma) were used for protein detection. Densitometry analysis was performed using the ImageJ software. Relative intensity for each experimental band was calculated by normalizing the absolute intensity of each protein with respect to the intensity of the corresponding β-actin band.

### 2.7. Statistical and Bioinformatics Analyses

Statistical analyses were performed using Student’s *t*-test. In all cases, probability *p*-values below 0.05 were considered significant. For in vitro data, at least two independent experiments were considered for the statistical analysis, unless otherwise indicated. For in vivo data at least five animals were considered for the statistical analysis, unless otherwise indicated. Fold change (FC) values were calculated as the ratio between average results in treated and control samples. The results are expressed as the mean ± standard deviation of three independent experiments.

### 2.8. Data Availability Statement

The materials used for the current study are available from the corresponding author C.A. (email: coambros@unisannio.it) or M.D.F. (email: mario.defelice@unina.it), on reasonable request.

## 3. Results

### 3.1. In Silico Toxicogenomic Analyses of Chlorpyrifos Activity Reveal a Gene Regulatory Network Potentially Involved in Thyroid Carcinogenesis

Previously conducted in silico toxicogenomic analyses of CPF activity, obtained by Ingenuity Pathways Analyses (IPA), revealed a gene regulatory network potentially involved in thyroid carcinogenesis.

**Figure 1:**
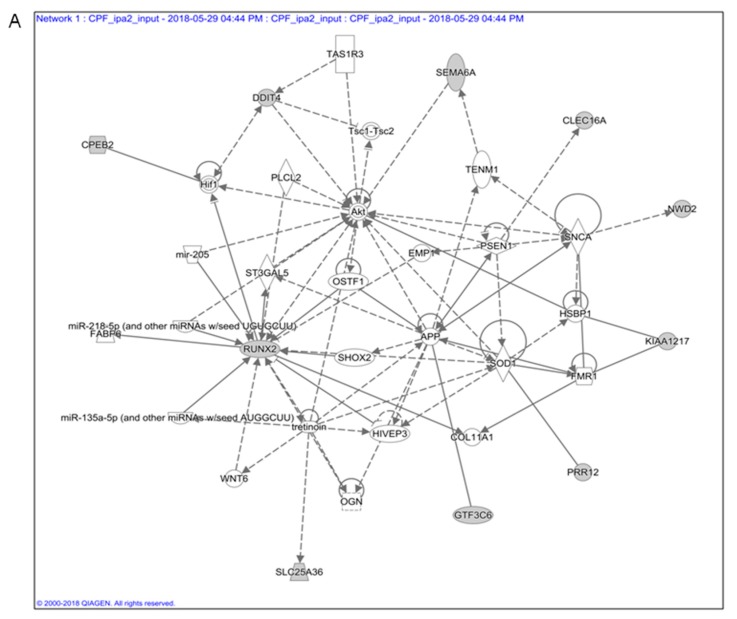

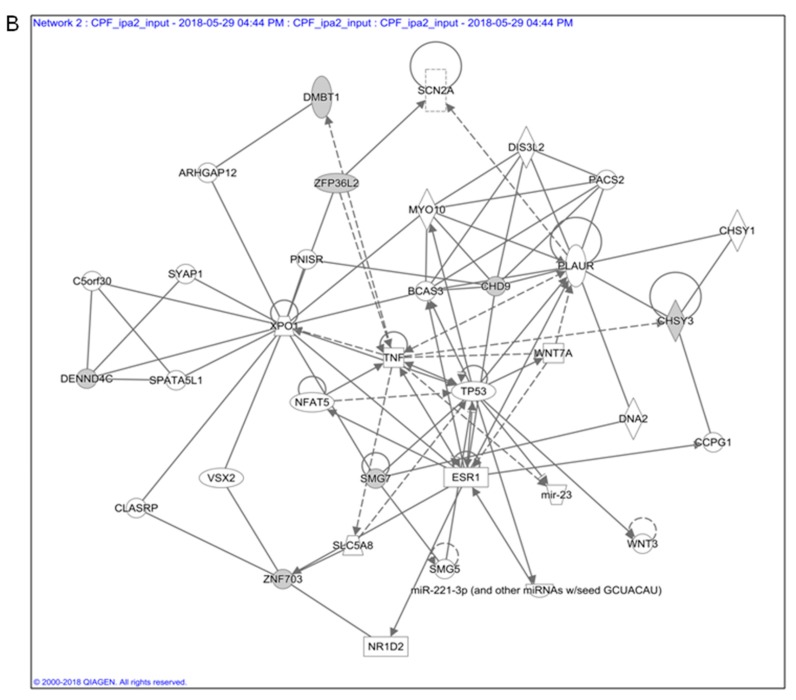
Network built up with the genes included in the endodermal carcinogenic gene signature (ECGS) derived from ToxoFunctions identified by Ingenuity Pathway Analysis (IPA) analyses. The differentially regulated genes (grey nodes) mapped to pathway annotations derived from literature and gene ontology using Ingenuity Pathway Analysis (IPA)). The solid lines connecting molecules represents a direct relation and dotted lines an indirect relation. IPA constructs networks that optimize for both interconnectivity and number of Focus Genes (the grey nodes) under the constraint of a maximal network size. White nodes are added by the algorithm to build a highly connected molecular network between Focus Genes.

**Figure 2:**
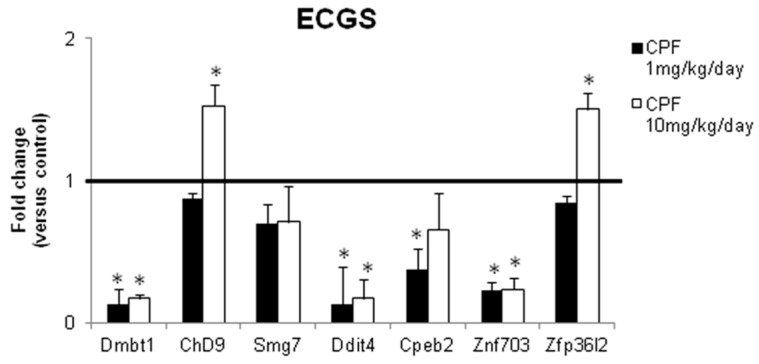
Life-long exposure to CPF regulates the expression of several mRNAs included in the ECGS and genes evidenced by the network analyses. mRNAs were prepared from thyroid of females exposed to 1 mg/kg/day and 10 mg/kg/day of CPF from the conception and life-long. They were sacrificed at 12 months and RNA prepared from thyroid. Level ECGS mRNAs, determined by real time polymerase chain reaction in control (not exposed) and treated animals. Data are reported as fold change values calculated as ratio between average relative gene expression in treated and control mice. Data are reported as the average and standard deviation of *Gapdh*-normalized mRNA levels of 5 animals. Mean and standard deviation is reported (Dmbt1 *p*-value = 0.02, *p* = 0.015; Chd9 *p* = 0.04; Ddit4 *p* = 0.025, *p* = 0.023; Cpeb2 *p* = 0.043; Znf703 *p* = 0.026, *p* = 0.035; Zfp36l2 *p* = 0.046).

**Figure 3:**
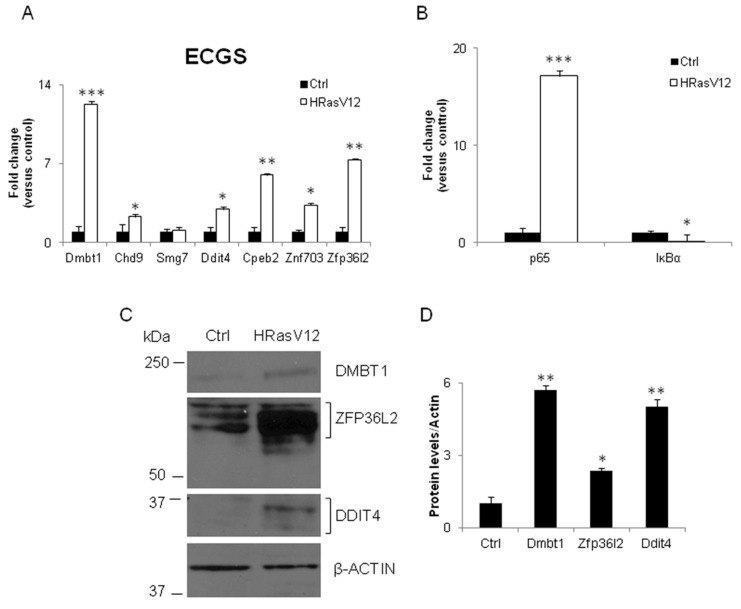
Chronic expression of constitutively activated RAS in immortalized thyrocytes induces several mRNAs included in the ECGS and genes evidenced by the network analyses. RNA was prepared from FRTL-5 cells transformed by mutated Ras (Ha-RasV12), expressing a constitutive Ha-RasV12 oncogene (FRTL-5-RasV12). (**A**) Level ECGS mRNAs, determined by real time polymerase chain reaction in control (FRTL-5) and transformed cells. (**B**) *p65* and *IκBα* mRNAs were determined by RT-qPCR in the same samples. Western blotting (**C**) and densitometry analysis (**D**) of DMBT1, ZFP36L2 and DDIT4 protein in FRTL-5 wild type and FRTL-5-RasV12 cells. β-Tubulin was used as loading control. Data are reported as fold change values calculated as ratio between average relative gene expression in treated and control cells. Data are reported as the average and standard deviation of *Abl*-normalized mRNA levels. Results are expressed as the mean ± standard deviation of three independent experiments. (Dmbt1 *p*-value = 0.0001; Chd9 *p* = 0.03; Ddit4 *p* = 0.035; Cpeb2 *p* = 0.003; Znf703 *p* = 0.034; Zfp36l2 *p* = 0.0054).

These genes belong to three networks implicated in neurological disease and carcinogenesis: (1) neurological disease, cell death and survival, developmental disorder (Figure 1A); (2) cancer, gastrointestinal disease, hepatic system disease (Figure 1B), (3) skeletal and muscular system development and function, cellular compromise, cellular function and maintenance (not shown).

The first two networks shared several genes, although only the second was associated to cancer. This network mainly refers to endoderm-derived organs, being associated mainly with liver disease and cancer. We explored the possibility that the same genes might be involved in thyroid carcinogenesis. Indeed, thyroid and liver are both derived by endoderm, and it has been proposed that mechanisms of cellular transformation by environmental carcinogens might be the same [27]. We selected 19 transcripts included in the Toxofunction Hepatocellular Carcinoma, Liver Hyperplasia/Hyper-proliferation, highlighted in Table 1. We grouped them in an ECGN. Several of the ECGN transcripts have a controversial role in cancer. Further analyses were conducted only on some of them, evidenced in italics in Table 1, whose role in cancer was evident in the available literature. They were included in an Endodermal Carcinogenic Gene Signature (ECGS). The detailed information regarding each investigated gene was reported in Supplementary Table S1.

Firstly, we verified the expression of the chosen transcripts of the ECGS in thyroid of mice exposed to CPF. We previously reported that 12 months exposure to CPF induced hypothyroidism in females and inhibited thyroid specific transcripts. We tested the level of *Ddit4*, *Zfp36l2*, *Zfp703*, *Dmbt1*, *Cpeb2*, *Chd9*, and *Smg7* mRNAs in thyroid of the same females, exposed since the conception and for 12 months to CPF (1 mg/kg/die and 10 mg/kg/die). As reported in Figure 2, the in vitro data were mainly validated in the in vivo model being *Dmbt1*, *Ddit4*, *Cpeb2*, *Znf703* transcripts inhibited in statistically significant manner. *Smg7* transcript showed a trend towards the inhibition. *Chd9* and *Zfp36l2* showed a different behaviour compared to the in vitro data being not regulated at the lower exposure doses and induced at the highest dose of CPF. Despite that, they were kept in the ECGS, considering their potential role in carcinogenesis.

### 3.2. Validation of the New Gene-Network in Cellular and Animal Models of Thyroid Carcinogenesis

Then, we verified the ECGS in cellular model of thyroid carcinogenesis. To this end, we tested the expression level of the ECGS transcripts in a cellular model of thyroid carcinogenesis represented by FRTL-5 cells expressing the constitutive Ha-RasV12 oncogene (FRTL-5-RasV12) [30]. RT-qPCR analyses were conducted on RNA prepared from parental FRTL-5 cells and chronically Ras-transformed cells (FRTL-5-RasV12). As shown in Figure 3A, surprisingly, all the analyzed transcripts were induced in FRTL-5 chronically transformed with mutated Ras, with *Smg7* being the sole exception. These cells do express very low levels of thyroid specific transcripts. We also assessed the expression of genes playing a key role in thyroid carcinogenesis as nuclear factor kappa-light-chain-enhancer of activated B cells (NF-ĸB) [34]. Its relevance was also underlined by the TNFα node in the network in Figure 1B. NF-ĸB activation was assessed by measure of the *IĸBα* mRNA, one of its targets that is also the main inhibitor of the pathway. *IĸBα* mRNA was inhibited, whereas the cellular content of *p65* transcripts, the transcriptional factor, was increased (Figure 3B). Both pointed to the activation of NF-ĸB pathway.

**Figure 4:**
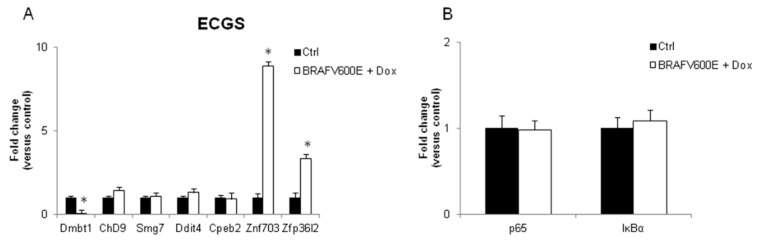
Doxycycline-induced expression of constitutively activated B-Raf proto-oncogene, serine/threonine kinase (BRAF) in vivo regulates several mRNAs included in the ECGS and genes evidenced by the network analyses. RNA was prepared from thyroid of mice expressing a mutated B-Raf oncogene only when treated with doxycycline (BRAFV600E + Dox) and from untreated animals (Ctrl). After 1 week of exposure, mice developed thyroid cancers and were sacrificed. (**A**) ECGS mRNA levels, determined by RT-qPCR in control and treated animals. (**B**) *p65* and *IκBα* mRNAs were determined by RT-qPCR in the same samples as in A. Data are reported as the average and standard deviation of *Abl*-normalized mRNA levels of 5 animals. Mean and standard deviation is reported. (Dmbt1 *p*-value = 0.030; Znf703 *p* = 0.015; Zfp36l2 *p* = 0.025).

We also verified the cellular content of some of the proteins codified by the ECGS. As expected, the cellular levels increased in the transformed cells (Figure 3C) concordantly with their transcripts, although the fold changes were not perfectly corresponding (Figure 3D). The ECGS mRNAs were also tested in a mouse model of thyroid cancer, represented by mice expressing a doxycycline inducible form of mutated B-Raf (BRAFV600E) [35]. The results shown in Figure 4A evidenced that only *Znf703* and *Zfp36l2* were significantly induced in thyroid cancer developed in mice by expression of oncogenic B-Raf, whereas *Dmbt1* was inhibited and the other transcripts not regulated. As reported in Figure 4B, *p65* and *IĸBα* were also not regulated.

**Figure 5:**
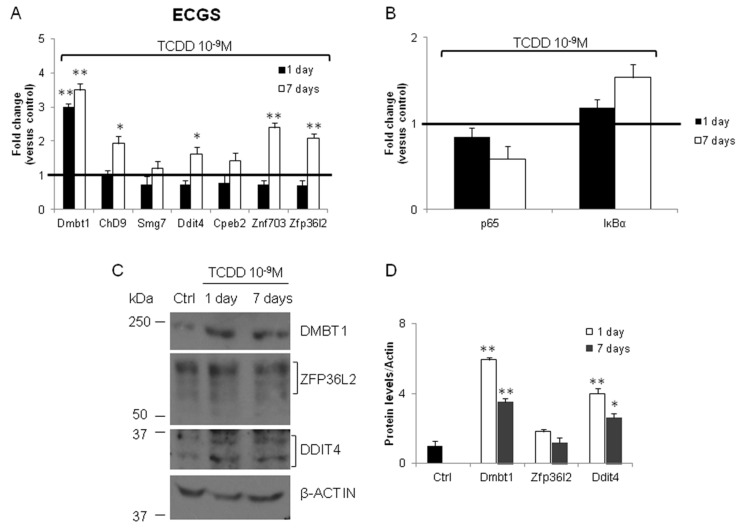
Tetrachlorodibenzo-*p*-dioxin (TCDD) exposure regulates the expression of ECGS transcripts in FRTL-5 in time-dependent manner. FRTL-5 cells were exposed to TCDD 10^−9^ M for 1 or 7 days or vehicle only (Dimethyl Sulfoxide, DMSO). RT-qPCR was performed as detailed in Materials and Methods and primers are listed in Table S1. (**A**) ECGS transcript levels in control and TCDD 10^−9^ M treated cells. (**B**) *p65* and *IĸBα* mRNAs were determined by RT-qPCR in the same samples. Western blotting (**C**) and densitometry analysis (**D**) of DMBT1, ZFP36L2 and DDIT4 protein in FRTL-5 untreated (Ctrl) and exposed to 10^−9^ M TCDD for 1 and 7 days. β-Tubulin was used as loading control. RT-qPCR data are reported as fold change values calculated as ratio between average relative gene expression in treated and control cells. Data are reported as the average and standard deviation of *Gapdh*-normalized mRNA levels. The black bar signs the control level. Results are expressed as the mean ± standard deviation of three independent experiments. (Dmbt1 *p*-value = 0.008, *p* = 0.0095; Chd9 *p* = 0.03; Ddit4 *p* = 0.042; Cpeb2 *p* = 0.018; Znf703 *p* = 0.01; Zfp36l2 *p* = 0.0075).

Epidemiological and experimental studies suggested the carcinogenic effect of TCDD. Therefore, we tested the expression of the same genes in FRTL-5 treated with TCDD (10^−9^ M) for 1 day or 1 week. As shown in Figure 5A, only *Dmbt1* mRNA was induced at the shorter exposure time. The *Ddit4*, *Zfp36l2*, *Zfp703*, *Cpeb2*, and *Smg7* transcripts showed a trend towards the inhibition. The cellular levels of *Ddit4*, *Zfp36l2*, *Zfp703*, *Dmbt1*, and *Chd9* were increased at the longer exposure time (Figure 5A). We also investigated the level of DMBT1, ZFP36L2, and DDIT4 by western blotting in the TCDD exposed cells (Figure 5C). The semi-quantitative analyses of the western blotting data (Figure 5D) pointed out that the trend toward the induction was evident and concordant with their transcripts for DMBT1 and DDIT4. The increase of the cellular content of ZFP36L2 protein was partially concordant with its transcript, suggesting that TCDD activity might act by different mechanisms in its regulation. We also evidenced a trend toward the induction for *IĸBα* (Figure 5B).

**Figure 6:**
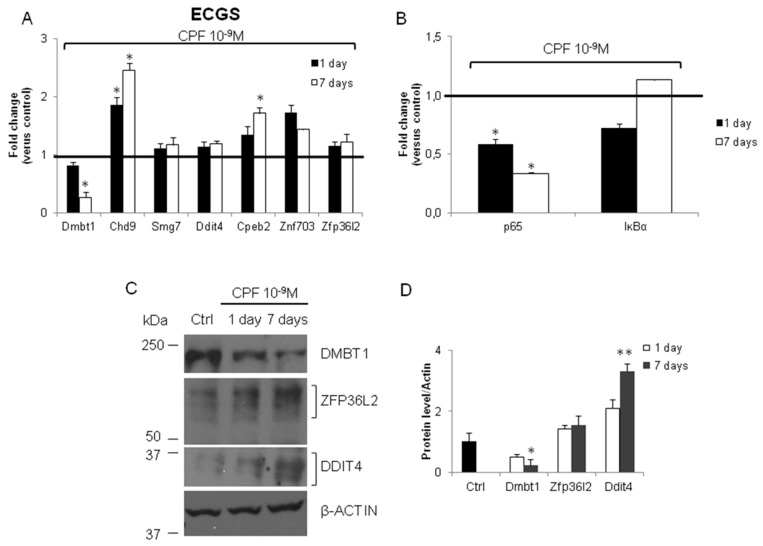
Chlorpyrifos exposure regulates the expression in FRTL-5 in time-dependent manner of ECGS transcript. FRTL-5 cells were exposed to CPF 10^−9^ M for 1 or 7 days or vehicle only (DMSO). RT-qPCR was performed as detailed in Materials and Methods and primers are listed in Table S1. (**A**) ECGS transcripts levels in control and CPF 10^−9^ M treated cells. (**B**) *p65* and *IĸBα* mRNAs were determined by RT-qPCR in the same samples. Western blotting (**C**) and densitometry analysis (**D**) of DMBT1, ZFP36L2 and DDIT4 protein in FRTL-5 untreated (Ctrl) and exposed to 10^−9^ M CPF for 1 and 7 days. β-Tubulin was used as loading control. RT-qPCR data are reported as fold change values calculated as ratio between average relative gene expression in treated and control cells. Data are reported as the average and standard deviation of *Gapdh*-normalized mRNA levels. The black bar signs the control level. Results are expressed as the mean ± standard deviation of three independent experiments. (Dmbt1 *p*-value = 0.049, Chd9 *p* = 0.021, *p* = 0.032; Cpeb2 *p* = 0.020).

Finally, we investigated the expression of ECGS mRNAs in the FRTL-5 exposed to CPF (10^−9^ M) for 1 day or 1 week. Indeed, they were evidenced in gene expression profiling experiment conducted on a different line of immortalized thyrocytes (PCCL3). As shown in Figure 6A, the main difference with the other in vitro models of thyroid carcinogenesis here investigated was the inhibition of *Dmbt1* mRNA, being *Chd9*, *Cpeb2*, and *Znf703* induced. Furthermore, we reported the inhibition of *p65* mRNA (Figure 6B). The investigation of the cellular content of DMBT1, ZFP36L2, and DDIT4 proteins was rather consistent with the level of their mRNAs (Figure 6C), even if the possibility of other mechanisms influencing the protein levels was evident (Figure 6D).

**Figure 7:**
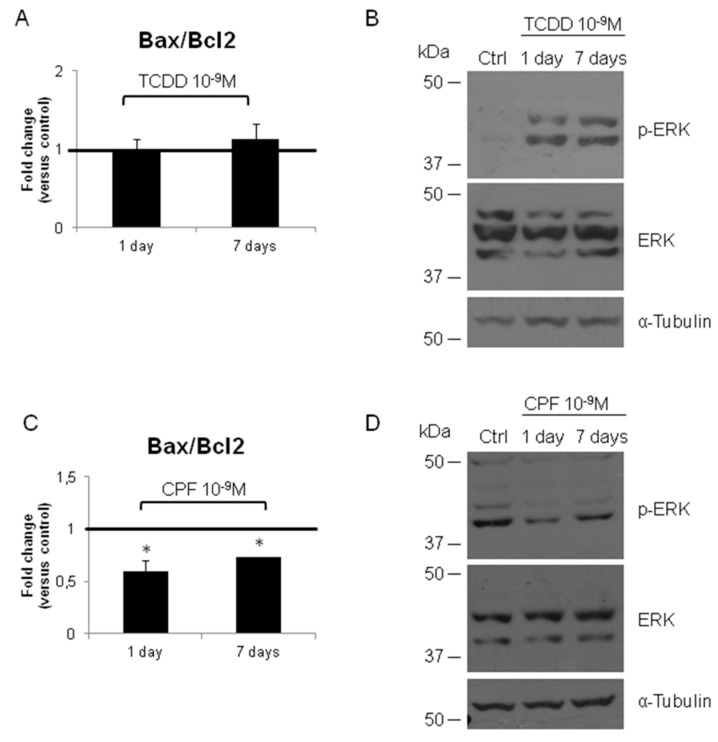
Tetrachlorodibenzo-*p*-dioxin (TCDD) and CPF exposure in FRTL-5 regulates the expression of genes involved in prevention of apoptosis and in mitogen-activated protein kinase (MAPK) signalling. (**A**) *Bax*/*Bcl2* transcripts ratio in FRTL-5 exposed to TCDD 10^−9^ M for 1 or 7 days or vehicle only (DMSO). RT-qPCR was performed as detailed in M&M and primers are listed in Table S1. (**B**) Western blotting analyses of MAPK activation in FRTL-5 treated with TCDD 10^−9^ M for 1 and 7 days, compared to untreated cells (Ctrl). pERK1/ERK2 was determined as well as the cellular content of ERK1/ERK2. α-Tubulin was used as loading control. (**C**) *Bax* and *Bcl2* transcripts ratio in FRTL-5 exposed to CPF 10^−9^ M for 1 or seven days or vehicle only (DMSO). (**D**) Western blotting analyses of MAPK activation in FRTL-5 treated with CPF 10^−9^ M for 1 and 7 days, compared to untreated cells (Ctrl). pERK1/ERK2 was determined as well as the cellular content of ERK1/ERK2. α-Tubulin was used as loading control. RT-qPCR data are reported as fold change values calculated as ratio between average relative gene expression in treated and control cells. Data are reported as the average and standard deviation of *Gapdh*-normalized mRNA levels. The black bar signs the control level. Results are expressed as the mean ± standard deviation of three independent experiments. (Bax/Bcl2, CPF *p*-value = 0.035, *p* = 0.048).

Considering the role that apoptosis plays in thyroid carcinogenesis, we investigate the level of *Bax* and *Bcl2* transcripts and the *Bax*/*Bcl2* ratio in FRTL-5 exposed to TCDD and CPF at different time points. As shown Figure 7A, TCDD did not alter the *Bax*/*Bcl2* ratio that, instead, was reduced by the CPF at both time points (Figure 7C). This suggests that CPF might promote cell survival. We also tested the activation of MAPK pathway in both conditions. As expected TCDD, the “true carcinogen” promoted activation of the pathway since the earlier time point analyzed (Figure 7B) whereas no detectable activation was induced by CPF (Figure 7D).

## 4. Discussion

Although still debated, the exposure to pesticides, as well as to other EDCs, has been associated with thyroid cancers in women [15,16]. This scenario opens up the possibility that environmental pollutants might promote thyroid cancer by direct and/or indirect action on thyrocytes. Here, we characterize on a molecular level the effects of a well-established environmental carcinogen, TCDD, and chemicals that are included in group 3 by International Agency for Research on Cancer (IARC) as CPF, recently suggested for further investigation [36]. Both these molecules are associated to hypothyroidism in human and rodents, a pathology also related to the increased incidence of liver and thyroid cancers [37,38]. This point is clinically relevant because it suggests that early life exposure to environmental EDCs, promoting subclinical thyroid disease, should be considered a risk factor for cancer development. Furthermore, it underlines the need to investigate common mechanisms, gene-networks of thyroid toxicity and carcinogenesis exerted by different environmental EDCs, poorly characterized until now. The reported data partially fulfil this gap, firstly investigating the CPF effects on immortalized rat thyrocytes [32] and identifying the gene-network associated with hepatocellular carcinoma further validated in other in vitro and in vivo model of thyroid cancers. We applied this gene network to thyroid cancers considering that: (i) IPA analysis refers to published results to build up the prediction and liver dysfunctions/carcinogenesis are widely investigated; (ii) we have recently proposed that organs of similar developmental origin might share common mechanisms of environmental carcinogenesis [26]; and (iii) liver shares the same developmental origin with thyroid. Despite the discrepancies in their regulation, the transcripts/proteins included in the ECGS or in the bioinformatically built ECGN were indeed altered in thyroid carcinogenesis in vitro (FRTL-5-RasV12 and FRTL-5 exposed to TCDD and CPF) and in vivo. Such discrepancies were observed between FRTL-5 and PCCL3 exposed to CPF, being the transcripts inhibited in the second in vitro model. The toxicogenomic data, obtained in PCCL3, are here validated by the results from the thyroid of CPF exposed animals (Figure 2). Our data confirm the already known differences between the two cellular models, since only the FRTL-5 is able to achieve a complete transformation upon infection with murine retroviruses; a second event is required for PCCL3. Overall, our data suggest that PCCL3 cell line is a better model to molecularly investigate the mechanisms of toxicity of an environmental pollutant by in vitro toxicogenomic whereas FRTL-5 modelled better thyroid carcinogenesis.

Modulation of cell proliferation, or surviving promotion, plays a pivotal role in endodermal carcinogenesis. The involved signalling pathways and gene-networks might be diverse and change differently along the time, making it conceivable that the impairment of certain cellular processes, or of the related gene-networks, is more relevant of the single gene activity. *Chd9* and *Dmbt1*, among the others, are part of several complexes/gene networks regulating cell cycle/apoptosis in the endoderm (Figure 1) and might be involved in thyroid carcinogenesis, a role not proposed until now. This suggestion is here confirmed. The results evidence their impaired expression in in vitro and in vivo models of overt thyroid carcinogenesis, such as in presence of constitutive activation of MAPK pathway, or exposure to environmental carcinogens, such as TCDD. Interestingly, they are similarly deregulated in cells exposed to CPF whose carcinogenic role in still discussed.

The opposite regulation observed in CPF exposed PCCL3 cells and the results obtained in thyroid carcinogenesis models could represent a weak point in the study. However, we should consider that the genes under investigation might behave as oncogenes in some cellular types/conditions and as onco-suppressors in some others. An example is *Dmbt1*, a gene usually deleted in brain cancers but induced in gastric precancerous lesions [39,40]. Accordingly, it is inhibited in thyroid cancer developed in BRAFV600E mice but induced in other conditions. Furthermore, they might promote carcinogenesis by multiple mechanisms. *Chd9*, for instance, might promote cancer development, inducing the transcription of genes involved in the assembly of ribosome [41] and of gene controlling invasiveness [42]. Another example is *Zfp36l2*, a transcript enriched in thyroid bud [43], member of a gene family codifying for post-transcriptional regulator factors, differently regulated in the in vitro and in vivo models here adopted. Its expression is increased in FRTL-5-RasV12 and FRTL-5 exposed to TCDD and CPF and this might be attributed to DNA replication defects/stress, also characterizing Ras-induced transformation of thyrocytes [44]. TCDD and CPF also act to promote DNA damage [45] and activate p53 pathway [46] that, in turn, might promote *Zfp36l2* gene expression. The reduction of *Ddit4*, a gene induced in stressing condition, is also consistent with this hypothesis, because its loss might promote cell proliferation [47]. Thus, CPF and TCDD- exposed FRTL-5 cells will be predisposed to develop an inadequate response to further stressors, as we have previously shown in the case of BPA, although other mechanisms were involved [5]. This last consideration implies that a different approach has to be undertaken in evaluating the effects of the exposure of compounds that cannot be directly genotoxic/carcinogenic but that can lower the response to second stressors.

The MAPK [23] pathways and cAMP signalling play a pivotal role in both events of thyroid carcinogenesis [22] whose first step is the promotion of apoptotic events followed by the loss of responsiveness to TSH and/or inactivation of apoptotic cascade in surviving cells.

Concordantly, our data evidence a dual role of NF-ĸB pathway, already suggested as regulator of apoptosis in thyroid [34]. Indeed, it is clearly induced in cells chronically expressing mutated Ras but not in cells exposed to TCDD or in thyroids of mice expressing BRAFV600E. In our opinion, the reported results are consistent with the need of both events for a complete thyroid cell transformation that, indeed, is not reached in vivo because the thyroid cancer mass usually is reduced upon doxycycline withdrawal. Instead, the lowering of *Bax/Bcl2* ratio in CPF-exposed cells, not coupled to MAPK activation, is suggestive of a prosurviving activity, also involved in thyroid carcinogenesis. This last point underlines a possible clinical relevance of our data because over-expression of the ECGN transcripts is associated to factors promoting thyroid cell proliferation by activation of MAPK pathways, whereas they are inhibited by factors promoting their survival. Thus, their analyses might identify thyroid cancers hopefully responsive to drugs promoting their apoptosis.

## 5. Conclusions

Considering the controversial behaviour of the single genes of the ECGS in carcinogenesis and their opposite regulation in comparison to gene expression profiling data, further studies are required to define their role in thyroid carcinogenesis. These investigations should be aimed to overcome the major limitations of this study consisting in the small number of models investigated and genes analysed. Nevertheless, we suggest their potential in molecular classification of cancer subtypes and as targets of novel therapeutic approaches.

The concomitant analyses of the ECGN transcripts can be relevant for the investigation of environmental promoted carcinogenesis. Their differential regulation in different carcinogenic settings points out that mechanisms of environmental-promoted carcinogenesis might be different, but, anyway, consisting in the promotion of cell surviving and proliferation, as in the case of TCDD. Regarding CPF carcinogenicity, the data suggest that it is a plausible indirect carcinogen, promoting conditions resembling a precancerous state characterized by impairment of the surviving rate more than the cell proliferation that can be worsened by a second stressor. Indeed, thyroid cells are constantly threatened by DNA damage due to both exogenous and endogenous stressors [3] and the weakening of DNA damage response might increase the rate of mutations. This should be considered as an alternative mechanism of environmental carcinogenesis. 

## Figures and Tables

**Table 1:** Ingenuity Pathway Analysis (IPA) biofunctions deregulated in chlorpyrifos (CPF) treated PCCL3. Biofunctions containing at least two genes are reported with indication of genes identifying each category. In bold are reported the genes included in the endodermal carcinogenic gene-network (ECGN).

Categories	Diseases or Functions Annotation	Corrected *p*-Value	Genes
Cell Cycle	Entry into G2 phase	2.86 × 10^−2^	EGR1, **RUNX2**
Cellular Growth and Proliferation, Embryonic Development	Formation of embryonic cells	2.86 × 10^−2^	GNAO1, **RUNX2**
Gene Expression	Transcription of DNA	2.86 × 10^−2^	BEX1, EGR1, FZD5, GTF3C6, HEXIM2, HMGA1, **RUNX2**, TARBP1, ***ZFP36L2***, **ZNF628**, ***ZNF703***
Cancer, Organismal Injury and Abnormalities	Epithelial cancer	2.86 × 10^−2^	BEX1, **C9orf172**, CDKL4, ***CHD9***, **CHSY3**, **CLEC16A**, ***CPEB2***, CTNNAL1, ***DDIT4***, **DENND4C**, ***DMBT1***, EGR1, FZD5, GNAO1, GTF3C6, HEXIM2, HMGA1, INPP5J, KIAA1217, **NWD2**, **PRR12**, **RUNX2**, SEMA6A, **SLC25A36**, **SMG7**, SUSD6, TARBP1, WDR76, ***ZFP36L2***, **ZNF628**, ***ZNF703***
Cellular Development, Hematological System Development and Function, Hematopoiesis	Erythropoiesis of cells	2.86 × 10^−2^	EGR1, HMGA1
Cancer, Organismal Injury and Abnormalities, Reproductive System Disease	Mammary tumor	2.86 × 10^−2^	BEX1, ***CPEB2***, CTNNAL1, ***DDIT4***, EGR1, GNAO1, INPP5J, **RUNX2**, SUSD6, ***ZFP36L2***, **ZNF703**
Cell-mediated Immune Response, Cellular Development, Cellular Function and Maintenance, Hematological System Development and Function, Hematopoiesis, Lymphoid Tissue Structure and Development	Differentiation of T-lymphocytes	2.86 × 10^−2^	EGR1, FZD5, HMGA1, **RUNX2**, ***ZFP36L2***
Cellular Development, Hematological System Development and Function, Hematopoiesis, Lymphoid Tissue Structure and Development	Development of lymphocytes		EGR1, Fus, FZD5, HMGA1, **RUNX2**, ***ZFP36L2***
Endocrine System Development and Function, Lipid Metabolism, Molecular Transport, Small Molecule Biochemistry	Secretion of aldosterone		EGR1, HMGA1

## Supplementary Materials

The following are available online at http://www.mdpi.com/1660-4601/16/1/122/s1, Table S1: CPF cluster genes, Table S2: Primer sequences used for RT-qPCR.
