# Supplementary material for: A Toxicogenomic Approach Reveals a Novel Gene Regulatory Network Active in In Vitro and In Vivo Models of Thyroid Carcinogenesis
Source: Int J Environ Res Public Health. 2019 Jan 4;16(1):122. doi: 10.3390/ijerph16010122 (PMC6338918; doi:10.3390/ijerph16010122)
Supplement: Supplementary file 1 [file ijerph-16-00122-s001.pdf]

Supplementary Material

A Toxicogenomic Approach Reveals a Novel Gene Regulatory Network Active in In Vitro and In Vivo Models of Thyroid Carcinogenesis

Carla Reale, Filomena Russo, Sara Carmela Credendino, Danila Cuomo, Gabriella De Vita, Massimo Mallardo, Francesca Pennino, Immacolata Porreca, Maria Triassi, Mario De Felice and Concetta Ambrosino

Table S1. CPF cluster genes.

Gene name	Gene ID	Gene ontology
Ddit4	140942	Cell proliferation
Zfp36l2	298765	Regulation of mRNA stability
Zfp703	680717	Regulation of cell cycle
Dmbt1	170568	Cell differentiation
Cpeb2	360949	Cellular response to oxidative stress
Chd9	307726	Chromatin organization
Smg7	360855	Regulation of RNA stability

Table S2. Primer sequences used for RT-qPCR.

Gene name	Species	Forward sequence	Reverse sequence
Gapdh	*R. Norvegicus*	CATGGCCTTCCGTGTTCCTA	AGCCTGCTTCACCACCTTCTTGAT
Ddit4	*R. Norvegicus*	TGGGATCGTTTCTCGTCCTC	TGGGATCGTTTCTCGTCCTC
Chd9	*R. Norvegicus*	CAAAGACGAAGGTGGTTTGAAGT	TTGGAGGTGTTCACAACGCT
Snrpb	*R. Norvegicus*	GAGAAGAGAAGCGAGTCCTTGG	GGGCAATGCCAGTATCTTTGG
Znf703	*R. Norvegicus*	CCATTGAGCTGGACGCCAAGA	GGCTCGGGTCTTTCTCGGAT
Dmbt1	*R. Norvegicus*	GGCTGGTAAATGGAACAAACCG	GGCACACGGTACCCAAGAG
p65	*R. Norvegicus*	CTTTCAAGGTGCCCTACTCGG	CACATGGCCAAAATCCACGG
IκBα	*R. Norvegicus*	CTTCTCCATCTTGCCTGTGA	CGTTGACATCAGCACCCAAA
Zfp36l2	*R. Norvegicus*	CCCCTCGTCCGTTATTCGTC	GGGATTTCTCCGTCTTGCACA
Bcl2	*R. Norvegicus*	CTGACGCCCTTCACCGCGAG	CAAAGGCATCCCAGCCTCCGT
Cpeb2	*R. Norvegicus*	GAGCACGATCCTCTCAAGGG	AAGATCGACCTCGTCTTCGC
Bax	*R. Norvegicus*	TTGCTACAGGGTTTCATCCAGG	CACTCGCTCAGCTTCTTGGT
Gapdh	*M. Musculus*	AGGTCGGTGTGAACGGATTG	TGTAGACCATGTAGTTGAGGTCA
Snrpb	*M. Musculus*	ATCGGGACCTTCAAAGCCTTT	CCCTTTCTGCTTGTTTGGAGTT
Znf703	*M. Musculus*	TCCTGCACCCGGAGTACCTG	TGGGTTAGTCCCCGAGTGTGAC
Dmbt1	*M. Musculus*	GTATTACTGCGAGAGGGTTCCA	GAATAGCCCATGGACTGAAGGT
Cpeb2	*M. Musculus*	GCTTGTTGGATGATGGTCACAG	ACCAAAGGTCCAAATCTTCGGA
Chd9	*M. Musculus*	CCCACTGTCTACCAACCATCAA	TTTCGTGGCTTCTTCTCAGACA
Bcl2	*M. Musculus*	ATGACTGAGTACCTGAACCGGCAT	GGGCCATATAGTTCCACAAAGGCA
IκBα	*M. Musculus*	CGCTCTTGTTGAAATGTGGGGG	ATAGGGCAGCTCATCCTCTGT
Ddit4	*M. Musculus*	GGGATCGTTTCTCGTCCTCC	ATGAGGAGTCTTCCTCCGGC
p65	*M.Musculus*	AAGCGCAAAAGGACCTATGA	GCTTGGGGACAGAAGTTGAG
Zfp36l2	*M.Musculus*	CCCTCGCCCGTTATTCATCT	CCAGGGATTTCTCCGTCTTG
Bax	*M.Musculus*	ACAGATCATGAAGACAGGGG	CAAAGTAGAAGAGGGCAACC
